# The state of the art of twinning, a concept analysis of twinning in healthcare

**DOI:** 10.1186/s12992-016-0205-5

**Published:** 2016-10-26

**Authors:** Franka Cadée, Marianne J. Nieuwenhuijze, A. L. M. Lagro-Janssen, Raymond De Vries

**Affiliations:** 1 CAPHRI School for Public Health and Primary Care, Maastricht University, P.O. Box 616, 6200 MD Maastricht, The Netherlands; 2 Research Centre for Midwifery Science, Zuyd University, Universiteitssingel 60, 6229 ER Maastricht, The Netherlands; 3 Research Centre for Midwifery Science Maastricht, Zuyd University, Universiteitssingel 60, 6229 ER Maastricht, The Netherlands; 4 Department of Primary and Community Care, Gender and Women’s Health, Radboud University Medical Center, P.O.Box 9101, 6500 HB Nijmegen, The Netherlands; 5 Center for Bioethics and Social Sciences in Medicine University of Michigan Medical School, North Campus Research Complex, 2800 Plymouth Road, Building 16, 419W, Ann Arbor, MI 48109-2800 USA; 6 CAPHRI School for Public Health and Primary Care, Maastricht University, Maastricht, The Netherlands

**Keywords:** Healthcare professionals, International collaboration, Midwives, Nurses, Physicians, Reciprocity, Twinning

## Abstract

**Background:**

Inequities in health have garnered international attention and are now addressed in Sustainable Development Goal 3 (SDG3), which seeks to ‘promote well-being for all’. To attain this goal globally requires innovative approaches, one of which is twinning. According to the International Confederation of Midwives, twinning focusses on empowering professionals, who can subsequently be change-agents for their communities. However, twinning in healthcare is relatively new and because the definition and understanding of twinning lacks clarity, rigorous monitoring and evaluation are rare. A clear definition of twinning is essential for the development of a scientific base for this promising form of collaboration.

**Method:**

We conducted a Concept Analysis (CA) of twinning in healthcare using Morse’s method. A qualitative study of the broad literature was performed, including scientific papers, manuals, project reports, and websites. We identified relevant papers through a systematic search using scientific databases, backtracking of references, and experts in the field.

**Results:**

We found nineteen papers on twinning in healthcare. This included twelve peer reviewed research papers, four manuals on twinning, two project reports, and one website. Seven of these papers offered no definition of twinning. In the other twelve papers definitions varied. Our CA of the literature resulted in four main attributes of twinning in healthcare. First, and most frequently mentioned, was reciprocity. The other three attributes were that twinning: 2) entails the building of personal relationships, 3) is dynamic process, 4) is between two named organisations across different cultures. The literature also indicated that these four attributes, and especially reciprocity, can have an empowering effect on healthcare professionals.

**Conclusions:**

Based on these four attributes we developed the following operational definition: *Twinning is a cross-cultural, reciprocal process where two groups of people work together to achieve joint goals*. A greater understanding and a mature definition of twinning results in clear expectations for participants and thus more effective twinning. This can be the starting point for new collaborations and for further international studies on the effect of twinning in healthcare.

## Background

Can twinning in healthcare play a role in sustainable development globally? At the United Nations General Assembly 2015 (UNGA) the 193 leaders adopted seventeen Sustainable Development Goals (SDGs) [1]. SDG 3 states that by 2030 we will *‘Ensure healthy lives and promote well-being for all at all ages.*’ In an era where the gap between rich and poor within and between societies is ever widening [2, 3], attaining SDG 3 will be a challenging task demanding a variety of collaborative approaches as stated in SDG17 [4]. Twinning is one of these approaches, a method that brings together health professionals across cultures in an effort to improve healthcare by empowering these professionals [5]. And indeed twinning does appear to have great potential for this purpose. Reports from the Swedish International Development Co-operation Agency (SIDA) describe twinning to have *‘potential advantages over other forms of development co-operation as it encourages organisational learning and sustainable development’* [6]. According to SIDA the potential of twinning has not yet been fully exploited. At present twinning in healthcare is implemented in a variety of ways ranging from straight forward linking of institutions or organising exchange visits to complex processes of bilateral development between healthcare professionals. This variation makes it difficult to pin down the essential features of twinning.

Historically twinning can be traced back to town twinning that was encouraged by the European Community for many decades following the Second World War. Twinning was defined as *‘a formal and substantive collaboration between two organizations and/or cities’* [7, 8]. It was intended as a way to improve international relations and reconciliation by fostering friendship and increasing understanding between different cultures and former foes [9]. Town Twinning caught on and was soon joined by twinning of various organisations in different settings from different countries. At the start, these activities ran in conjunction to Town Twinning but they soon functioned independently to the point that we now have twinning going on all over the globe in many different shapes and contexts, with examples ranging from twinning between diabetes associations of Mozambique and the UK to improve diabetes care [10], the Tonga twinning Program between hospital staff of Australia and Tonga [11] to “toilet twinning” between any two toilets worldwide to improve world sanitation [12].

If twinning is to play an important role globally in empowering healthcare professionals to perform to their potential [7, 13], further scientific studies need to be done to underpin assumptions of effectiveness. Without definitional clarity and a full understanding of the concept, it is difficult to assess the value of twinning or to discover which aspects of twinning contribute to its success. In this paper we offer a concept analysis (CA) of twinning in healthcare using a critical exploration of the literature where *‘the literature is used as data’* [14], aiming for an operational definition and a mature understanding of the concept twinning in healthcare.

## Method

Given the lack of definitional clarity, we performed a CA to gain a clearer view on the definition and key attributes of twinning as a type of international collaboration. Table 1 gives an explanation of the CA terminology used in this article.

**Table 1:** Concept analysis terminology

Concept Analysis terminology	
Concept	Conceptual representations of a phenomenon
Attribute	A characteristic of a concept that appears over & over again
Boundary	What is & what is it not part of the concept (delimiting cases)
Variation	A similar term with *some* overlapping attributes
Consequence	The effect brought about by attributes of a concept

### Morse’s method of concept analysis

We chose Morse’s method [14] for our CA because it offers a useful framework whereby the pragmatic utility of a concept can be established taking into account the dynamic context in which the concept is used. According to Morse, concepts can either be emerging or mature. A mature concept is well defined, has clearly described attributes, delineated boundaries and documented preconditions and outcomes. The degree of maturity determines the usefulness of the concept [14]. In order to use concepts in research, they need to be mature. For this reason it is necessary to evaluate the level of maturity of the concept twinning [15].

### Applying Morse method to twinning in healthcare

**Fig. 1:**
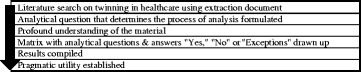
Process of Concept Analysis according to Morse

Figure 1 describes the steps we use to conduct our CA.

Between December 2014 and August 2015 we did a literature search using Ebscohost (Psycinfo, Cinahl), Embase, Medline, Pubmed and Web of Science (medical/social sciences). This search and selection was done by the author in close collaboration with the second author. The key search terms were ‘healthcare professionals’, ‘twinning’ and ‘international’ (Table 2). The search was limited to papers published from 1985 – when digitalisation of Medline began—to 2015, with no language restrictions. All papers that mentioned the term twinning and healthcare professionals in the title and/or the abstract were selected. The full articles were then obtained and read and any that did not deal with twinning as a form of collaboration were discarded. To extend our search, we hand searched the reference lists of included articles and consulted experts in the field. As all literature is seen as data in a CA, a rich sample including discussion papers, final project reports and manuals pertinent to twinning were also included.

**Table 2:** Search strategy for CA on twinning

Search strategy
Midwi* OR Nurse* OR Health personnel OR
Healthcare workers OR Doctor* OR Physician*, AND
Twinning, AND
International OR Intercultural OR Cultural

Subsequently, we used an extraction document to collect the main characteristics for each paper, such as dates, definitions, assumptions, boundaries, contexts (such as country), variations, attributes, type of healthcare professionals, source of funding and quotations to facilitate the discussion. The maturity of the concept twinning in healthcare was assessed using the indices: definition, attributes, boundaries, preconditions and consequences [15]. As the understanding of the data deepened analytical questions we formulated and reformulated several times. The three final analytical questions used to distil attributes and boundaries from the data were:

1. What attributes make twinning distinctly different from other forms of collaboration?
2. Is reciprocity a core value of twinning?
3. Are the other emerging attributes & boundaries present in all papers?

The extraction document was used to tabulate the answers to question 1. This data was then rigorously analysed and incorporated in a matrix shown in Table 3. This matrix allowed oversight and critical analyses of the literature helping to clarify the concept and develop an operational definition by distilling the attributes, boundaries and consequences from the data. We then used the matrix to answer questions 2 and 3 with ‘Yes’, ‘No’ or ‘ Exception’ when the answer was unclear. The data extraction and analysis was primarily done by the first author but was scrutinised by all authors.

**Table 3:** Definitions & attributes of twinning from the literature

			Attributes				
Author	Health care professional	Definition of twinning	Based on reciprocity or similar	Building personal relation-ships	Between two cultures	Is a dynamic process	Boundaries examples
[19] AIHA, 2010	mixed	a partnership that links two entities with shared characteristics to achieve a common goal’	Y sharing	Y peer relationships	Y	Y	–
[20] Breiddal, 2009	mixed	‘a professional and social collaboration between organizations in different countries to achieve mutual benefits through combined efforts and a common vision’	Y reciprocity	Y developing friendships with like-minded people	E Canada sub-Sahara Africa	Y	fundraising by ‘Western’ partner, provide one way assistance
[21] Busse, 2013	mixed	‘ when two or more academic institutions or community organizations share collective knowledge and resources to address issues and concerns’	Y reciprocity	Y builds collective efficacy	Y Ethiopia & USA	Y	–
[23] Cadée, 2013	midwives	methodology of mutual exchange between different organisations	Y reciprocity	Y being like sisters	Y Netherlands & Sierra Leone	Y	–
[28] Chiu, 2005	nurses	none	N	E	Y Malaysia & Australia	N	obtaining bachelor degree, one way visit
[24] Dawson,2014	midwives	partnerships through pairing organisations	Y mutual learning & ownership	Y immersing each other’s practice	Y	E	–
[29] Foster, 2013	nurses	none	Y mutual respect and dialogue	Y compassion fatigue	Y USA & Dominican Re-public	Y	–
[48] Hopkins, 2013	nurses, physicians	two-way transfer of expertise, advice, knowledge and skills	Y mutual respect from both sides of the partnership is crucial.	N	Y	Y	–
[18] ICAD, 1999	nurses, physicians	uses WHO 2001 definition: a formal substantive collaboration between two organisations’	Y a 2 way process, win-win	Y	N Canadian & different ‘southern’ partners	Y	North/South connections
[5] ICM, 2014	midwives	a two-way mutually beneficial exchange between two member Midwives Associations. It is a formal and substantive collaboration between two organisations’[7]	Y collaborative relationships, mutual learning	Y being like sisters (no big or little sister)	Y Canada & Tanzania, Sierra Leone & The Netherlands Mali & Switzerland Papua new Guinea & Australia Japan & Philippines	Y	–
[16] Foster, 2009	physicians	none	E	E	E	E	matching organisations
[25] Ireland, 2015	midwives	none	Y reciprocity	Y	Y Nepal & England	E	one way help
[30] Jiang, 2015	nurses	A collaborative relationship between 2 similar organizations.	Y mutual respect	Y	Y China & USA	Y dynamic cooperative atmosphere	funding by ‘Western’ partner
[17] Kohi, 2010	nurses	none (matching)	?	N	Y Tanzania & US	N	matching organisations
[22] Macdonagh, 2002	mixed	The establishment of a formal link between a specific department/ institution in the UK and a corresponding department/institution in the developing world	Y mutuality	E	Y Tanzania & UK	Y	help from a developed country
[33] Qaddoumi, 2009	physicians	none	Y mutual relationship	E	Y Jordan & Canada	Y	help from a developed country
[26] RCM, 2015	midwives	none	Y reciprocity and mutuality	Y sisterhood	Y England & Nepal Scotland & Uganda Wales & Cambodia	E	–
[49] Herschderfer, 2012	midwives	two midwifery organisations and 40 midwives are linked with the joint aim of improving maternal health, basing the relationship on reciprocity	Y reciprocity	Y Being like sisters	Y Netherlands & Sierra Leone Netherlands & Morocco	Y	–
[32] Veerman, 2005	physicians	twinning is an established form of cooperation between resource rich and resource limited countries.	E	N	Y Indonesia & The Netherlands	E	–

*Y* yes, present, *N* not present, *E* Exception

## Results

**Fig. 2:**
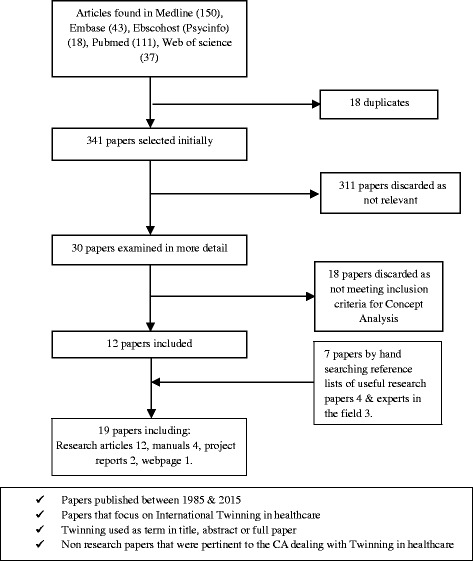
Literature review process for CA on Twinning in healthcare

Of the 359 hits in the search, 341 remained after excluding double papers. We excluded a further 311 papers based on the title and the abstract, as they did not meet our inclusion criteria. Many of the excluded papers were about congenital twins. Subsequently, after fully reading the remaining 30 papers, 12 papers were selected which met all the criteria. We found an additional 7 papers by hand searching the reference list of selected papers and by consulting two experts on twinning in healthcare. These were manuals and reports that were not available in the scientific databases but relevant for a concept analysis of the broad literature, leaving us with 19 articles in total as shown in Fig. 2.

Table 3 shows the Matrix with characteristics of the nineteen selected papers on twinning in healthcare.

Twelve papers were peer reviewed research articles and two were final project reports of twinning projects. The four manuals and one web page were written to guide different healthcare professionals in twinning collaborations. Using the indices on the maturity of the concept twinning according to Morse, the data showed twinning to be an emerging concept with competing definitions, lacking clarity and without clearly identified attributes, boundaries and consequences.

### Definitions of twinning in healthcare

Seven of the nineteen papers had no definition of twinning. Two of these seven papers used the term twinning as an adjective meaning ‘matched’ [16, 17]. In the remaining twelve papers twinning was used as a verb indicating a process. Two papers used the definition from the WHO City Twinning Guide [7]: *‘twinning is a formal substantive collaboration between two organisations’* [5, 18]. Even though this definition originates from organisations of international standing, the definition has not been integrated into the broader literature or research on twinning in healthcare. The definitions in the other nine papers varied and lacked clarity. All definitions incorporated some type of collaboration by using terms such as partnership, collaboration, exchange, sharing, pairing, cooperation, linking or exchange.

### Participants in twinning

Of the nineteen papers, six described twinning projects in mixed (care and cure) healthcare teams where the nurses, were generally included in a later phase of the project and the implementation of the project as well as reporting was in the hands of physicians [18–22]. Six papers describe twinning between midwives [5, 23–27], four between nurses [17, 28–30] and two between physicians. In the data on twinning projects with nurses and midwives the four emerging attributes as described below appeared more frequently than in the data with physicians where delimiting cases appeared more frequently demonstrating the boundaries of twinning.

### Attributes of twinning in healthcare

Four distinctive attributes of twinning were appeared over and over, clarifying our understanding of the concept. The four attributes we identified are that twinning:

1. is based on reciprocity,
2. entails the building of personal relationships,
3. is a dynamic process,
4. is between two named organisations across different cultures.

As twinning can only be defined by the distinguishing attributes, we did not include other commonly mentioned attributes such as learning, lasting for a substantial period of time, having a formal contract, being culturally sensitive, being (internally or externally) funded, developing leadership skills, exchanging ideas, knowledge or technology are not included as they are characteristic for many forms of collaboration and therefore not specific for twinning [31].

1. **Twinning is based on reciprocity** ***:*** The term reciprocity used in this paper is defined as *‘exchanging things with others for mutual benefit’* (Oxford dictionary 2003). Fourteen papers mention reciprocity as a core value of twinning suggesting it to be a defining feature of twinning programs. Reciprocity is either used explicitly or implicitly with terms like “mutual”, “2-way” or “sharing” (see Table 3). Characteristic quotes of reciprocity as a core value of twinning are:
*‘In situations where economic disparity exists, a conscious effort needs to be made to ensure that the Twinning relationship and activities are approached as equal partners.’* P10 [5]
*‘…building a twinning partnership is grounded in shared guiding principles that emphasize the importance of building long-term relationships…based on equality, reciprocity, shared responsibility.’*P1312 [21]
*‘Specifically, the dynamic cooperative atmosphere fosters learning through the sharing of respective experiences’* P118 [30]
*‘The most successful twinning projects are those that are two-way – i.e. each organization learns from the other. Two-way twinning is empowering to both organizations.’*P25 [18]
2. **Twinning entails the building of personal relationships:** Twelve papers speak of the importance of building a personal relationship between twins. For example, twins in twinning projects between midwives are described as *‘being like sisters’*, nurses and physicians talk of *‘developing friendships with like-minded people’* or *‘being immersed in each other’.* Additional quotes exemplify the importance of personal relationship:
  ‘ *You must be open to examining your own attitudes and behaviour. Reflect honestly on your willingness to wait and watch (when appropriate), to learn from your twinning partner, to refrain from “fixing” and giving advice, and to become aware of assumptions or prejudgments*.’ P5 [20]
*‘Despite the language challenges and 7300 miles that separate our 2 facilities, nurses speak a common language.’* P121 [30]
3. **Twinning is a dynamic process:** A similar pattern emerged in the literature with references to putting the accent on the twinning process with room for spontaneous development. Eleven papers mentioned this, using terms such as: *‘flexibility’, ‘dynamic cooperative atmosphere’, ‘synergy’* or ‘*collective efficacy’.*
4. **Twinning is between two named organisations across different cultures:** All twinning projects in the selected papers occurred between two similar organisations, such as two hospitals [21], two hospices [20] two training facilities [29] or two associations of health professionals [23, 25]. Five of these papers specifically mention people to people twinning within these organisations [21, 23, 27, 30]. Six definitions specifically mention that twinning takes place between two organisations. Three papers [20, 22, 32] mention that twinning takes place between organisations from two different countries. In all other papers it is assumed that the relationship is between two named organisations in two different countries from, for example, Ethiopia and USA [21], England and Nepal [25] or Jordan and Canada [33]. From the data it becomes clear it is not so much the national border, but the difference in culture that is a requisite for twinning.

### Boundaries of twinning in healthcare

Several papers use the concept twinning but don’t include the above attributes. These cases give us a clear view of the boundaries of twinning. This can be demonstrated by two of the papers which use the concept twinning as an adjective to mean ‘matched’ [16, 17], one paper that describes a case study of twelve Malaysian students to investigate why they want to do their studies abroad [28] and one paper specifically mentions a one way, rich to poor, process *‘it gives young paediatricians from the less affluent nations a chance to improve their skills’P106* [32]*.* In these last two papers the term twinning appears to be misplaced as it is one sided development and lacks the key attributes we describe above.

Of all nineteen papers, three describe twinning between the physicians, six in mixed teams and ten between the nurses and/or midwives (Table 3). The attribute of building a relationship is mentioned less frequently by those papers that discuss twinning between physicians [16, 32] or mixed teams [22, 33]. Building a personal relationship is less clear or absent when twinning is interpreted as ‘matching‘ [16, 17] or as a strategy for obtaining a Bachelor’s degree abroad [28]. Quotes that illustrate this boundary are:

*’The demand for a degree for Malaysian nurses provided the impetus for this offshore post-registration nursing programme, developed as a twinning venture between an Australian university and a Malaysian private institution.’*P46 [28]

*‘The goal was to ensure that nursing students would have entry-level knowledge and skills in the care and treatment of people living with HIV.*’P93 [17]

### Consequences of Twinning in healthcare

In none of the nineteen papers used in this CA was the impact, or the consequences, of twinning systematically investigated by the authors. Our examination of the literature, however, uncovered a probable correlation between reciprocity and the empowerment of healthcare professionals. Empowerment is taken to mean *‘a powerful catalyst for positive change’* [34, 35]. Thirteen authors make a point of emphasising the importance of reciprocity to achieve empowerment. Authors specifically mentioned this at least once under either a special heading [5, 18, 23, 24, 26, 27], or in the introduction [20, 29], the conclusion [21, 22, 25] or abstract [19, 30]. The following quotes illustrate the emphasis that authors put on reciprocity for empowerment.

*‘Specifically, the dynamic cooperative atmosphere fosters learning through the sharing of respective experiences….twinning requires the implementation of shared strategies and developmental goals.’* P118 [30]

*‘Recognising that the UK has much to learn from low-income countries, the meaning and process of true exchange, reciprocity and mutuality should be further explored.’* P5 [26]

‘*Reciprocity is an important aspect of twinning as the RCM also hopes to strengthen midwifery in the UK.‘*p26 [25]

In the remaining five papers empowerment is not mentioned. In these papers twinning is seen as matching [16, 17] or the emphasis is on a specific end result such as obtaining a bachelor degree [28] or the training of healthcare professionals [32, 33].

## Discussion

### Defining twinning in healthcare

The literature on twinning in healthcare to date has been as diverse as its definitions. Analysing the literature using Morse’s CA methodology led to the identification of four attributes that distinguish twinning from other types of international collaboration. These are that twinning 1) is based on reciprocity, 2) entails the building of personal relationships, 3) is a dynamic process, 4) is between two named organisations across different cultures. The data indicate that these four attributes, but foremost reciprocity, relate to empowerment. Empowerment can therefore be seen as a consequence of twinning. If twinning in healthcare continues without the use of a clear definition, enthusiasm about its potential will remain mere enthusiasm and the outcomes will be no more than the outcome described in one of the first papers written on twinning in healthcare ‘we all learned a great deal’ [36]. As a result of this CA the following definition can be formulated using the emerged attributes: *Twinning is a cross-cultural reciprocal process where two groups of people work together to achieve joint goals.*

### The maturity of the concept twinning

The concept twinning in the literature to date is broad and used as an everyday concept similar to its use in common language, its function being to facilitate communication of an abstract activity in daily life. Why was the concept twinning used in this way? We think that twinning as a concept has certain connotations that makes it appealing to use. After all, the innate congenital meaning of two separate beings that are born at the same time stirs the imagination. This makes twinning very visual and gives marketing value to the concept. This positive reputation of twinning may be the reason why the term to date has been used so freely.

In conducting this CA we have deepened our understanding of twinning and contributed to the maturity of concept (Morse}. The analysis of the data has brought a clear consensual definition, with clearly described attributes and boundaries. Empowerment has emerged as a consequence (effect) of twinning. Other potential outcomes however cannot yet be fully described or demonstrated until the definition is integrated in the field and the outcomes are monitored.

### Reciprocity in twinning in light of the Gift theory

The first three attributes of twinning, that it 1) is based on reciprocity, 2) entails the building of personal relationships, 3) is a dynamic process, together with their interconnectivity can be better understood using the classic work of Mauss on ‘the gift’ [37]. While his work describes ancient societies, it remains relevant in 2016 and clearly demonstrates why reciprocity is an essential feature of twinning.

The importance of reciprocity for successful twinning emerged clearly from the data, yet most authors reported difficulty in bringing theory into practise because of problems with checks and balances. In Mauss’s Gift theory three aspects to gift giving are described: that of the giver, the receiver, and the reciprocator, which assumes social interaction. This interaction is reflected in the attributes two and three of twinning. According to Mauss *‘The unreciprocated gift still makes the person who has accepted it inferior, particularly when it has been accepted with no thought of returning it’ *[37]. This aspect of gift giving is also used in the critique on top down, North-South, post-colonial development aid [38–41], the main critique being that*‘…they offer aid to promote autonomy whilst buying influence for themselves’* [40]. The debt of the receiver is described as disempowering [42]. In twinning the attribute of reciprocity is described by authors as a core value. A consequence of the giving process is what Kowalski calls a *“useful tension that will ultimately have to be released by a response at an appropriate moment”* [40], it is therefore not surprising that authors mention the difficulty in balancing power relationships. The tripartite system of giving–receiving–reciprocating is described as a continuous process and participants of twinning have to take on each of these three roles in turn. The challenge of balancing their power relationship is an integral part of this process, asking for continuous (re)assessment. This also suggests that twinning is not a quick win but a process that needs to be given time. The ancient yet familiar approach of reciprocity to circumnavigate the potential disempowering effect of developing aid is an innovative aspect of twinning.

According to Gift theory, the giver does not give with the aim to receive a gift in return, *‘the giving of a gift proclaims the desire for a relationship’* [43]. This aspect emerged in the data as the second attribute of twinning, the building of a personal relationship. The sustainability of twinning appears to be directly related to this relational investment made by both ‘twins’. This is not only a personal investment, but also one of time and finances. Several authors mention equal funding to be an integral part of the twinning process [5, 27]. This warrants a new approach by donors because all twins, also those from higher income countries, will benefit.

### Twinning and empowerment

Empowerment is seen an important strategy for the reduction of health inequities [44]. Most authors in this CA mention empowerment as an effect of twinning especially with reference to the attribute of reciprocity. There are many definitions of empowerment with contradictions and tensions including a discussion of whether empowerment is a goal or a means to an end. There is critique of its normative power *‘carrying the allure of optimism and purpose’* as well as that it has become a *‘Buzz word’* [45]. With reference to the empowerment of women, feminist critics say the term can be used *‘to keep donors happy’* [46]. Keeping this critique in mind, most definitions do imply a strong positive value where persons gain control over their life [35]. The definition used in this paper – ‘a powerful catalyst for positive change’ – includes most of the characteristics mentioned as well as being true to the comments made in the papers used for this CA. The link found between reciprocity and empowerment is not a surprise, as reciprocity directly addresses the inequality between participants as discussed in light of the Gift Theory. Development aid often struggles with unequal power relations, as noted by Berry: ‘*Working toward empowerment to reduce these inequities inevitably means addressing power relations and resource distribution* [34]*.* Twinning deals with this issue upfront by embracing reciprocity as a core value.

### Other observations

The international/cultural aspect of the fourth attribute, that twinning is between two named organisations across cultures, can partly be the result of the search terms used. However, the attribute of ‘engaging two named organisations, or people within those organisations’ emerged from the data itself and is a legitimate attribute of the concept of twinning.

Boundaries of twinning such as matching, helping, one sided funding, giving training (one sided) and some characteristics of medical tourism indicate that some of the projects described in the data can be better described as training programmes [16, 17, 32] or study tours [28]. That absence of the four attributes of twinning and the consequence of empowerment in these articles confirm these findings.

We found that nurses and midwives use twinning as a form of collaboration more often than physicians or mixed teams. In mixed teams, the physicians were in the lead and the nurses often included later in the programme. The attributes of building a relationship and seeing twinning as a process instead of a means to an end was mentioned more often in twinning by nurses and midwives and to a lesser extent by physicians and mixed teams. This may indicate a gender difference because, despite the shift that is taking place, most nurses and midwives in the data were still women and most physicians men. Sex of the authors of the papers confirms this. This discrepancy between nurses, midwives and physicians could be because women tend to value the importance of personal relationships and the process more than men [47] who tend to be more oriented to outcomes. However different goals or other contextual issues could also be of influence. These, as well as the gender aspects of twinning, warrant further investigation.

### Limitations

It is difficult to ascertain if all literature on twinning was included in this CA because it might not have been categorised as twinning, just as some literature on twinning included here should have been categorised as exchange or other forms of international collaboration. The latter option is more likely to have been the case because the immaturity of the concept twinning meant that the term was used freely to describe many different types of international collaboration.

Two of the papers included in this CA were written by one of the authors creating a possible bias concerning the outcomes of this CA. The authors were aware of these limitations and were alert in not allowing this to intervene with the results. Excluding congenital twins using search terms was impossible because it resulted in the exclusion of some twinning collaborations. The large number of hits on congenital twins was easy to differentiate and therefore did not pollute the results. They were excluded by hand. Table 3 reflects the information about the projects that was accessible in the published articles.

## Conclusion

Integrating a new definition of twinning in practise clarifies the expectations of this method for participants, a necessary step for the successful implementation of twinning and for evaluating the outcomes of twinning programs. Effective twinning has an empowering effect, which, in turn, equips healthcare professionals to play their part in SDG 3 [13]. Because twinning empowers both twins, it is an innovative answer to the critique that development aid promotes dependency. The challenge of attaining equality that is often experienced in twinning is an integral part of its dynamic reciprocal process. Because this process warrants continuous monitoring, (re)evaluation and adjustments, twinning is not a ‘quick win’. Twinning also has the benefit of encouraging funders to revisit their vision for development: with its focus on reciprocity and equality between partners, it demands a change in standard practices.

Our new definition of twinning is an important first step in the development of this form of international collaboration, allowing it to be distinguished from others ways of working together that are more appropriately categorised as exchange, cooperation, or training programmes. Furthermore, the clear definition we offer here will facilitate new research, creating a scientific basis for twinning that will provide a richer understanding of how to better use this method.
